# Round the Hospitals

**Published:** 1919-10-18

**Authors:** 


### ROUND THE HOSPITALS.

Among honours for war services it is announced that the King has been pleased to award the Royal Red Cross (First Class) to Miss J. M. Cruickshank, Acting Matron Q.A.I.M.N.S.R. In a list of foreign honours granted at various dates to the British Forces we note the name of Miss R. Osborne, M.B.E., R.R.C., Acting Principal Matron Q.A.I.M.N.S., who received from the President of the French Republic the Médaille des Epidémies (en vermeil).

**ROUND THE HOSPITALS**—(*continued*).

Much interest attaches to the Army Order, which we reprint on page 62, relating to the pensions and gratuities awarded by the War Office to nursing sisters disabled during the war. The Order was issued in September 1917. On July 31, 1919, a letter was sent from the College of Nursing to the Minister of Pensions, calling attention to the position of nurses who have served the country during the past five years and are now out of employment, and to the fact that many are in great distress, broken in health, mentally or physically, and will never again be able to follow their profession. In response to this letter attention was drawn by the authorities to the Army Order issued under a Royal Warrant in 1917. The regulations made two years ago lay down a scale of disablement pensions to nurses and of temporary and permanent awards; nurses are to be assisted to undergo treatment, and are to receive grants during treatment; grants are also to be made to disabled nurses to help them to take training for other avocations; gratuities are to be given for minor forms of disablement, and are also available under certain conditions for disablement neither caused nor aggravated by military service. The chain of assistance would appear complete.

Unfortunately the Army Order thus issued appears to be wholly unknown, not only to the majority of demobilised nurses in need of assistance, but even to those most intimately concerned in relieving their distress. Not only so, but many nurses state that they have given up applying to the Ministry of Pensions, because they get no answer to their applications. If this be really the case, which sounds hardly credible, it is evident that a serious hitch has occurred in putting into practice the admirable regulations, which were intended to safeguard nurses who had suffered injury of any kind to their health during their war work. The regulations were drawn up while war was in progress, and may have worked very well in the case of women who were in the service of the War Office at the time when their disablement occurred. In other cases the Nation's Fund for Nurses has been enabled to step in, and over and over again has rescued from something like destitution women who have deserved well of the State. This matter will continue to receive our attention. It needs remedy without further delay.

No one should visit Scotland who takes an interest in hospitals without making it their business to call at the Western Infirmary, Glasgow, and ask the medical superintendent and matron to be kind enough to afford them facilities to go through the whole institution and make themselves familiar with the thorough way in which the work in every department is conducted and continuously pursued. The question of nurses' hours has engaged the full attention of the matron, Miss Gregory Smith, with the support of her superintendent and committee. The arrangements in regard to the nursing staff are still in progress, but they have already reached a stage when, by making an increase in the number of nurses employed, it is made possible to give the nurses on day duty a 56-hours' week. In practice this works out at an average of eight hours per diem, exclusive of the annual holiday.

For the reasons stated in the leading article in the *Nursing Mirror* of last week it is not always possible to have a hard-and-fast eight hours day for nurses. This is manifest to those engaged in the administration of hospitals of any size, where the pressure on the beds is continuous, and the wards are fully occupied. No hospital can arrange a system whereby a nurse can be stopped in the middle of bandaging a limb, or hand over the taking of a temperature when it is proceeding, or when engaged in the operation-theatre in the middle of an operation, on the ground that she has been eight hours on duty, and the minute for her departure from it has struck. Nurses who take up nursing in the proper spirit have no desire to be regarded as automatic machines, nor will they consent to desert their patient until everything is so arranged that the patient can suffer no injury nor run any risk by the nurse's sudden withdrawal from duty.

It may be interesting to mention in this connection that the authorities of the Royal Infirmary, Glasgow, are making arrangements to adopt the principle of a fifty-six hours week. Both these hospitals are worthy of a visit. The time occupied in their inspection will be well spent, owing to the fact that many up-to-date and important points of administration will be met with, giving rise to thought concerning new developments in other hospitals of the first rank.

An event of great interest will be held on October 22, when a luncheon will be held in the Balmoral Rooms of the Trocadero Restaurant in honour of the ladies who have directed "the uniformed women auxiliary services" during the war. Princess Alice, Countess of Athlone, will preside. Last October a similar occasion took place in honour of the Matrons-in-Chief of the Military Nursing Services of the Crown, including those from the Dominions. This year Lady Ampthill, as director of the Voluntary Aid Detachments, Dame Florence Leach, head of Q.M.A.A.C., Dame Katharine Furse, head of the Women's Royal Naval Service, Mrs. Gwynne-Vaughan, head of the Women's Royal Air Force, Miss Muriel Talbot, of the Women's Land Army, and Dr. Flora Murray, of the Endell Street Hospital, are among the honoured guests. An influential committee has charge of the arrangements.

An increase in the uniform allowance for military nurses has long been overdue. It is now announced that new members of the Q.A.I.M.N.S., the Reserve, and the T.F.N.S. will receive £20 for uniform allowance for the first year, £5 for the second,

**ROUND THE HOSPITALS—*(continued)*.**

and £10 for each subsequent year, while all serving members will receive for the future, £10 a year for upkeep grant. The V.A.D. nursing members and the special military probationers are to receive £4 10s. each six months, the amount of refund being increased to £2 5s. Increases take effect from September 18, 1919, and will last until prices are materially reduced. Nearly all the civil hospitals have been compelled to increase their uniform grant when they do not supply materials. In the latter case, the increases in cost is some 100 per cent.

Nursing sisters and aides serving with the Q.M.A.A.C. in sick bays in France under conditions stated in Army Council Instruction 423, are now eligible for the bonus given to nurses retained on military service. It will be remembered that this bonus is as follows: Staff-nurse, 8s. 9d. a week; sister, 10s. 6d.; assistant-matron and matron, 17s. 6d.; principal matron, 24s. 6d.; matron-in-chief, 28s. 6d.; V.A.D. members are eligible for a grant of 3s. 6d.

We learn with much satisfaction that our sanguine anticipation that the new certificate would not be denied to nursing sisters whose names are mentioned in despatches has been realised. A communication from the War Office Publicity Department announces that all nurses who have had the honour of being "mentioned" will receive a certificate of the King's appreciation of their services, provided they are mentioned for work in France, Egypt, or other places overseas.

The Ministry of Health is taking prompt measures to reduce the scarcity of practising midwives. It is well known that the women who are willing to serve the rural population in this branch of work are not able to pay the high fees commanded by midwifery schools, nor are the voluntary organisations which labour to subsidise them capable of meeting the demands made on their funds. A new White Paper has been issued defining the rules under which grants in aid of midwifery training can now be procured. They are to be paid not to the students themselves, but in the form of scholarships to institutions which give approved courses of instruction. Grants not exceeding £20 will be paid for each student who declares her *bona fide* intention to practise as a midwife. Health visitors who have either been three years in full-time employment or have completed a recognised course of training will also be eligible for this assistance.

The Women's Police have won the confidence of the public during the short time they have been in existence to a quite remarkable extent. Their record of work accomplished week by week in London and other towns is for the most part secret history. So many young girls rescued from dangers they hardly guess at and placed under welfare supervision; so many unstable folk either cautioned or safely shepherded to their own homes; so many women and children helped, comforted, and relieved in difficulties too numerous to specify; a solid achievement, for it helps to keep prisons empty and homes together. It is interesting to know that many trained nurses have been attracted to this work, which affords golden opportunities of using professional knowledge to great advantage, and is of a nature to call forth the full powers, mental, moral, and physical, of those who set their hand to it.

Dr. H. Sutherland has been telling us that under present circumstances we are all eating too little butter, and we can but ruefully agree with him. His assertion that Holland could send us hundreds of tons of butter, were she allowed to do so by the Food Controller, does not conduce to contentment under restrictions. But, on the other hand, we have the reiterated statement of Mr. Roberts that there is a world shortage of butter owing to war, and no surplus stocks anywhere. The increase of tuberculosis during the war has so many other plausible explanations that Dr. Sutherland fails to convince when he lays it on deficiency of "cow butter." How much of this delicacy was ever obtainable by the labourers' family in pre-war days? We seem to remember school feasts, both in London slums and country districts, at which bread and butter was the favourite course, because never tasted except on such occasions.

The League of Roses in connection with the Great Northern Central Hospital has raised £6,550 since its inception nine years ago, and the managers have decided to name a ward after it in recognition of the services rendered by members. Lady Moore distributed badges and bars to over 200 members on October 9, each of whom had collected 10s. or more during the year.

The *Daily News* has been telling its readers that the hospital nurse offers a striking example of the disastrous effect produced by "following a profession," and a kind reader and member of the College of Nursing calls our attention with just indignation to the strictures passed on nurses in general in this connection. "Give her the slightest opportunity," the critic declares, "and a nurse will launch forth into the most vivid and sometimes bloodcurdling descriptions of her various cases." Nothing could be more unjust than a sweeping statement of this kind. The bloodcurdling nurse will perhaps be always with us, but we recognise her as a neurotic who has mistaken her profession rather than been perverted by it. Nurses do sometimes fail to realise that what is ordinary routine to them has a painful effect on their hearers, but those who talk gruesome "shop" at the dinner-table are happily very rare.

The annual report of the Nightingale Fund for 1918 is unusually interesting as marking the close of the war period. The two Nightingale Sisters, Miss Eborall (Mrs. Rigold) and Miss Rogers, paid for by the Fund, continued at the Brigade Hospital at Etaples until its destruction by bombs

**ROUND THE HOSPITALS**—(*continued*)

from German aeroplanes, and fortunately were not among the injured. Fifteen honours were received by Nightingale nurses during the year, and we confess we should have liked to see the full list published. The concessions made by the Nightingale Fund to V.A.D. nursing members and military probationers are very substantial. They are received as pupils, if suitable, in the Nightingale Home free of payment, on the same terms as though they were paying probationers, the fee of £5 for the preliminary training being remitted, as well as the fee of £30, while they will be entitled to receive their certificate at the end of three years, and not be bound to serve the hospital for the fourth year. The scholarship scheme adopted in 1914 and postponed during the war is, as our readers know, now in operation, three certificated students being in residence from the Nightingale School at King's College for Women, where they are qualifying to become sister-tutors.

Sir Henry Burdett's advocacy of the pay-ward system in hospitals all over the country has been brought afresh before the public in an interview published by the *Observer*. As an expert on all matters relating to hospitals, he has long held the opinion that in this direction lies the remedy for the depressing financial outlook of many voluntary institutions, and that the step is urgently demanded by the middle classes. A correspondent, "K. K.," who is a private nurse, writes to ask what is to become of the private nurses if pay-wards are instituted? In our judgment Sir Henry Burdett is consulting the best interests of nurses in advocating this reform. The great blot on our nursing system is the over-work and under-payment of our private nurses. If "K. K." will refer to our recent Foreword on the Eight-Hour Day for Private Nurses, she will see that paying wards are suggested as the best, and indeed the only, remedy for the hardships inflicted on nurses when, owing to want of means, patients who need two or more nurses are permitted to wear down the strength and undermine the health of one devoted woman. Many of the hardships from which private nurses suffer are due to the poverty of the patients, who cannot properly afford home treatment, but will not have recourse to charitable institutions.

Managers of institutions should not fail to attack any instance of profiteering brought under their notice either on the part of contractors or private firms. Out of carelessness and easy-going belief in the honesty of everyone concerned, many institutions pay far higher than they need for common necessaries, as may be seen by contrasting the sums paid by the different hospitals for milk, meat, groceries, and other supplies. In making complaints it is only necessary to bring the circumstances to the notice of the Secretary of the Complaints Committee, Profiteering Act Department, 54 Victoria Street, S.W. 1. The complainant must give the date of the transaction, the conditions under which it was entered into, the names and addresses of the parties, the precise nature of the goods, and the price paid or demanded. As a public duty none who have evidence of profiteering ought to shrink from taking action.

In connection with the proposed reconstitution of the Central Midwives Board, the effect of which will be to increase the membership from nine to twelve, the Midwives Act Committee of the London County Council urges that advantage should be taken of the opportunity to press for representation of the County Council on the Board. The Committee point out that under the provision of the Midwives Act, 1918, any deficiency in the accounts of the C.M.B. is apportioned between the councils of the several counties and county boroughs in proportion to the population. Upon this basis the L.C.C. is called upon to bear over 12 per cent. of the adverse balance, so that the financial aspect of the Board's transactions is a matter of concern to the County Council. As regards questions relating to the practice of midwifery, it is pointed out that the County Council has had great experience in matters arising under the Midwives Acts, having regard to the number of midwives in London.

The Tramway Committee of Glasgow Corporation has now considered a proposal suggested by the Scottish Midwives Association for facilities for registered members of that Association to travel on tramcars which are already carrying more than the statutory number of passengers. There was a keen debate, and eventually the committee, by 13 votes to 8, granted the application, and so Glasgow midwives will always be assured of a place in a tramcar, even when it is full up.

The death of Mrs. Kelly, aged 77, at Stoke Newington, recalls a tragic chapter of history. Mrs. Kelly was a sister-in-law of the late Lord Brampton, and was in Belgium with her son, Mr. Hawkins, in November 1914, when this gentleman was arrested under suspicion of being a spy. This was a great shock to her, and it would have gone hard with her mind had she not been rescued and assisted to escape by Miss Edith Cavell. Since that time Mrs. Kelly has shown symptoms at times of being mentally unhinged. She died suddenly of heart failure, following bronchitis.

The Health Committee of the Essex County Council has considered a claim from Midwife E. E. Millett for two guineas, being the fees she would have obtained from two maternity cases upon which she could not attend owing to being suspended from practice. Under Section 6 of the Midwives Act, 1918, the local supervising authority by whom a midwife is suspended may, if it thinks fit, pay her such reasonable compensation for loss of practice as under the circumstances seems just. The Committee recommends the payment of the claim.

